# ALKBH1L Is an m^6^A Demethylase and Mediates PVY Infection in *Nicotiana benthamiana* Through m^6^A Modification

**DOI:** 10.3390/plants14243796

**Published:** 2025-12-13

**Authors:** Jue Zhou, Shuocong Sun, Jingtao Yuan, Li Dong, Xinhua Wang, Chenchen Jing, Muhammad Amjad Nawaz, Ruimin Tang, Hui Cao, Bihua Nie, Xue Feng

**Affiliations:** 1 Shanxi Key Laboratory of Integrated Pest Management in Agriculture, College of Plant Protection, Shanxi Agricultural University, Taiyuan 030031, China; m19858002636@163.com (J.Z.); s630549595@163.com (S.S.); yuanjingtao2025@163.com (J.Y.); m15163152497@163.com (L.D.); wxh13920753409@163.com (X.W.); jccdyhm@sina.com (C.J.); liusq71@163.com (H.C.); 2 Higher Engineering School of Agrobiotechnology, National Research Tomsk State University, Lenin Ave, 36, Tomsk 634050, Russia; m.a.nawaz.cabb.uaf@gmail.com; 3 Centre for Research in the Field of Materials and Technologies, National Research Tomsk State University, Lenin Ave, 36, Tomsk 634050, Russia; 4 College of Life Sciences, Shanxi Agricultural University, Taigu 030801, China; tangrm@126.com; 5 National Key Laboratory for Germplasm Innovation and Utilization of Horticultural Crops (HZAU), Key Laboratory of Potato Biology and Biotechnology (HZAU), Ministry of Agriculture and Rural Affairs, College of Horticulture and Forestry Science, Huazhong Agricultural University, Wuhan 430070, China

**Keywords:** m^6^A, demethylation, *Potato virus Y*, NbALKBH1L, cylindrical inclusion

## Abstract

N^6^-methyladenosine (m^6^A), the most prevalent internal mRNA modification in eukaryotes, is also present in plants and is known to influence plant–virus interactions. However, its specific role in regulating *Potato virus Y* (PVY; *Potyvirus yituberosi*) infection, a major pathogen of potatoes, remains unclear. This study identified 16 potential m^6^A regulator genes in *Nicotiana benthamiana* through homology screening of *Arabidopsis thaliana* AlkB family members. Based on expression profiles in leaves at various developmental stages and following PVY infection, NbALKBH1L was selected for further analysis. Enzyme assays confirmed its m^6^A demethylase activity. Experiments with NbALKBH1L mutants, using RT-qPCR and m^6^A-IP-qPCR, demonstrated that it regulates PVY infection via the m^6^A pathway. Further investigation revealed that NbALKBH1L interacts with the PVY-encoded cylindrical inclusion (CI) protein. An interaction network constructed through immunoprecipitation–mass spectrometry (IP-MS) and RNA sequencing (RNA-seq) suggested that NbALKBH1L may serve as a central node in plant antiviral immunity, potentially linking metabolic processes with the regulation of viral infection. In summary, this study advances our understanding of plant m^6^A modifications in antiviral defense and provides valuable insights for future antiviral breeding strategies.

## 1. Introduction

RNA molecules are essential carriers of genetic information and play key roles in regulating various biological processes. Like DNA and proteins, RNA also undergoes multiple chemical modifications that affect RNA metabolism, determine RNA fate, regulate gene expression, and enrich the diversity of RNA functions and genetic information [1,2].To date, more than 160 types of chemical modifications have been identified in RNA molecules from archaea, bacteria, and eukaryotes, including m^7^G, m^1^A, m^6^A, m^6^Am, m^5^C, hm^5^C, Nm, I, Ψ, ac4C, and others [3]. The m^6^A modification involves the methylation of the N^6^ position of adenosine in RNA molecules. This modification was first discovered in Novikoff hepatoma cells in 1974 and is currently considered the most abundant and conserved RNA modification [4,5,6]. The components involved in m^6^A methylation modification include methyltransferase complexes, demethylases, and reader proteins: the methyltransferase complex catalyzes the formation of the m^6^A modification, functioning as the “writers”; demethylases act as “erasers”, removing the methyl group from mRNA. The combined action of writers and erasers maintains m^6^A in a dynamic and reversible balance; m^6^A methylation reader proteins serve as “readers”, primarily responsible for recognizing RNA modifications and executing subsequent biological functions. Together, these three components coordinate to regulate and determine the fate of RNA [7,8,9,10,11,12,13].

In plants, current research on m^6^A has primarily focused on its effects on mRNA 3′ UTR processing [14,15], stability [15,16], and translation efficiency [17,18]. In contrast, its roles in miRNA biogenesis [19], RNA nuclear export, and alternative splicing [20] have been less frequently reported. The study of m^6^A modifications in plants began as early as 1979 when Kennedy and Lane first identified this modification in wheat [21]. However, its significance in epigenetics remained largely unrecognized for decades due to technological limitations in sequencing and the prevailing perception of RNA base modifications as static and irreversible. These constraints significantly hindered the advancement of research in this area. In mammals, fat mass and obesity-associated protein (FTO) was established as an m^6^A demethylase, demonstrating for the first time that m^6^A modifications are dynamically reversible in vivo. This discovery revealed that the regulation of m^6^A parallels that of DNA and histone methylation, suggesting its significant biological functions [8]. In plant m^6^A research, studies have revealed that approximately two-thirds of *Arabidopsis* mRNAs contain m^6^A modifications. This discovery enabled the identification of mRNA adenosine methylase (MTA), a core methyltransferase that localizes primarily to reproductive organs, apical meristems, and nascent root hairs. Subsequent studies demonstrated that homozygous *MTA* knockout mutants exhibit embryonic lethality, confirming its essential role in plant growth and development [22]. A homolog of *Arabidopsis* MTA, designated PvMTA, was identified in common bean (*Phaseolus vulgaris* L.). Functional analyses through the overexpression and silencing of *PvMTA* demonstrated its negative regulatory role in the response to *Bean common mosaic virus* (BCMV; *Potyvirus phaseovulgaris*) infection. Overexpression of *PvMTA* increased the levels of m^6^A modification on BCMV genomic RNA, promoting the degradation of viral RNA and thereby suppressing BCMV infection [23]. In *Arabidopsis*, ALKBH10B regulates m^6^A modifications on the transcripts of genes such as FT, SPL3, and SPL9. This regulation influences their mRNA degradation rates and consequently controls flowering time [24]. Yu et al. heterologously overexpressed FTO in rice and potato, resulting in significant yield increases in both transgenic crops. These findings demonstrate the potential of m^6^A demethylases to enhance plant yield and provide new insights for future crop breeding [25]. Relevant studies indicate that *Arabidopsis ECT2*, *ECT3*, and *ECT5* loss-of-function mutants exhibit increased susceptibility to *Alfalfa mosaic virus* (AMV; *Alfamovirus AMV*), with significantly elevated viral accumulation. Furthermore, knocking out *ECT2*, *ECT3*, and *ECT5* in the background of the m^6^A demethylase *ALKBH9B* knockout mutant restores the virus’s ability to establish systemic infection. These findings suggest that ECT proteins function as key effector molecules in m^6^A-mediated antiviral immunity [26]. Research has demonstrated that under saline stress, the expression of *ECT8* is upregulated, which increases its binding to m^6^A-modified mRNAs. This process accelerates the expression of salt-tolerance genes, thereby enhancing plant tolerance to salinity [27]. Additionally, m^6^A modification plays a crucial role in the plant–virus arms race. Studies have revealed a bidirectional antagonistic mechanism centered on m^6^A modification in plant–*Cucumber mosaic virus* (CMV; *Cucumovirus CMV*) interactions. The viral coat protein recruits host m^6^A methyltransferases to catalyze m^6^A marking of viral RNA; this mark is recognized by the ECT8 reader protein, which promotes viral RNA degradation and restricts infection. Conversely, the viral 2b protein suppresses viral m^6^A deposition by binding to the methyltransferase subunits MTB and HAKAI, while also globally reducing host m^6^A levels and disrupting the expression of defense-related genes [28]. In summary, m^6^A modification serves as a crucial regulatory mechanism that governs plant growth, development, and responses to stress.

The genus *Potyvirus* represents the largest taxonomic group among plant-infecting viruses, encompassing more than 200 officially recognized species to date [29,30]. *Potato virus Y* (PVY; *Potyvirus yituberosi*), a member of the genus *Potyvirus* (family *Potyviridae*), serves as the exemplar species for this taxonomic family [29,31]. PVY possesses a positive-sense, single-stranded RNA genome of approximately 9.7 kb. The genomic RNA features a 5′-terminal viral protein genome-linked (VPg) and terminates in a 3′ polyadenylated tail [32,33,34]. Post-translational cleavage of the viral polyprotein by PVY-encoded proteases (P1, HC-Pro, and NIa-Pro) generates ten functional proteins, with the P3N-PIPO protein additionally produced through ribosomal frameshifting [35]. PVY exhibits broad host specificity, infecting 163 plant species across 34 genera—primarily within the Solanaceae family—where it ranks among the most devastating pathogens of potato (*Solanum tuberosum*). Significant economic losses also occur in *Nicotiana tabacum* and species within the *Asteraceae* species [36,37,38]. Yue et al. analyzed virus-infected samples using MeRIP-seq technology and found m^6^A modification sites in both the coding and non-coding regions of the PVY genome. Following the downregulation of the ALKBH9 homolog in *Nicotiana benthamiana*, the accumulation of PVY was significantly reduced, providing evidence that m^6^A modification plays a crucial role in the viral infection process. However, the direct mechanism by which the AlkB protein functions in this process remains unclear [39].

In this study, we identified NbALKBH1L, an m^6^A demethylase in *N. benthamiana*. We demonstrate that NbALKBH1L reacts to PVY infection and associates with the viral cylindrical inclusion (CI) protein within the cytoplasm. Our findings suggest that NbALKBH1L likely modulates viral infection via m^6^A-dependent regulatory mechanisms. Furthermore, we conducted integrated immunoprecipitation–mass spectrometry (IP-MS) and RNA sequencing (RNA-seq) analyses to globally identify proteins that interact with NbALKBH1L in response to viral infection and to delineate their biological functions. This multi-omics approach established a comprehensive interaction network for NbALKBH1L, providing a critical dataset for mechanistic exploration. Intriguingly, the characterization of NbALKBH1L knockout lines revealed its potential involvement in root development, suggesting pleiotropic regulatory functions that extend beyond viral defense. Collectively, these findings position NbALKBH1L as a multifaceted m^6^A regulatory component that orchestrates complex biological processes in plants through integrated molecular networks.

## 2. Results

### 2.1. Identification of AlkB Gene Family Members, Gene Domains, and Analysis of Physicochemical Properties in N. benthamiana

**Figure 1:**
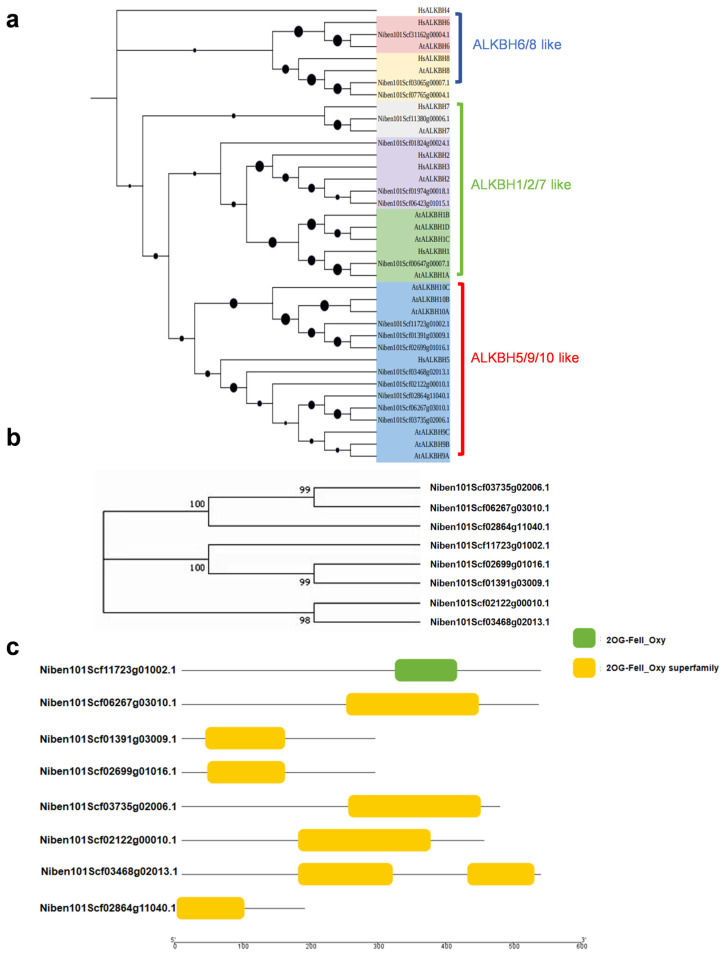
Analysis of the ALKB family of *Nicotiana benthamiana*. (**a**) Homologous evolution analysis tree of NbALKBH1L and AtALKBH9B; (**b**) Evolutionary analysis tree of *N. benthamiana* m^6^A demethylase candidate genes; (**c**) Analysis of the conserved structural domains of the ALKB family of *N. benthamiana*, green boxes represent the 2OG-Fell_0xy domains, while orange boxes represent 2OG-Fell_Oxy superfamily domains.

To find m^6^A demethylase enzyme genes in *N. benthamiana*, we examined its genome using fourteen known AlkB family members from *A. thaliana* as reference sequences. This bioinformatics analysis revealed Sixteen orthologous AlkB genes (Figure 1a). The domain analysis using CD Search eliminated genes without functional domains, leaving us with sixteen candidate genes. We then performed a phylogenetic analysis to compare these genes with the fourteen AlkB family members from *A. thaliana*. *A. thaliana*, where ALKBH9 and ALKBH10 are orthologs of the human demethylase HsALKBH5. Both AtALKBH9B and AtALKBH10B have been demonstrated to possess m^6^A demethylase activity in vitro and in vivo [24,40]. Therefore, the eight *N. benthamiana* genes that clustered with AtALKBH9/10B are potential candidates for m^6^A demethylases, likely sharing a similar demethylation mechanism (Figure 1b). To further characterize the structural features of the putative m^6^A demethylases in *N. benthamiana*, we conducted phylogenetic and domain analyses on these eight candidate genes. All encoded proteins were found to contain either the 2OG-Fe(II) oxygenase superfamily domain, or the specific 2OG-Fe(II) oxygenase domain, indicating a high level of conservation of these domains (Figure 1c). Finally, we analyzed the physicochemical properties of these genes and predicted their subcellular localizations. Their protein lengths, relative molecular masses (MWs), isoelectric points (pIs), and subcellular localization results are presented in Table S1. The protein lengths ranged between 192 and 540 amino acids (aa), with molecular weights varying from 22,015.31 Da to 61,855.06 Da, and pI values falling within the range of 5.61 to 9.37.

### 2.2. Expression Patterns of m^6^A Demethylase Candidate Genes in PVY Infected N. benthamiana Leaves

**Figure 2:**
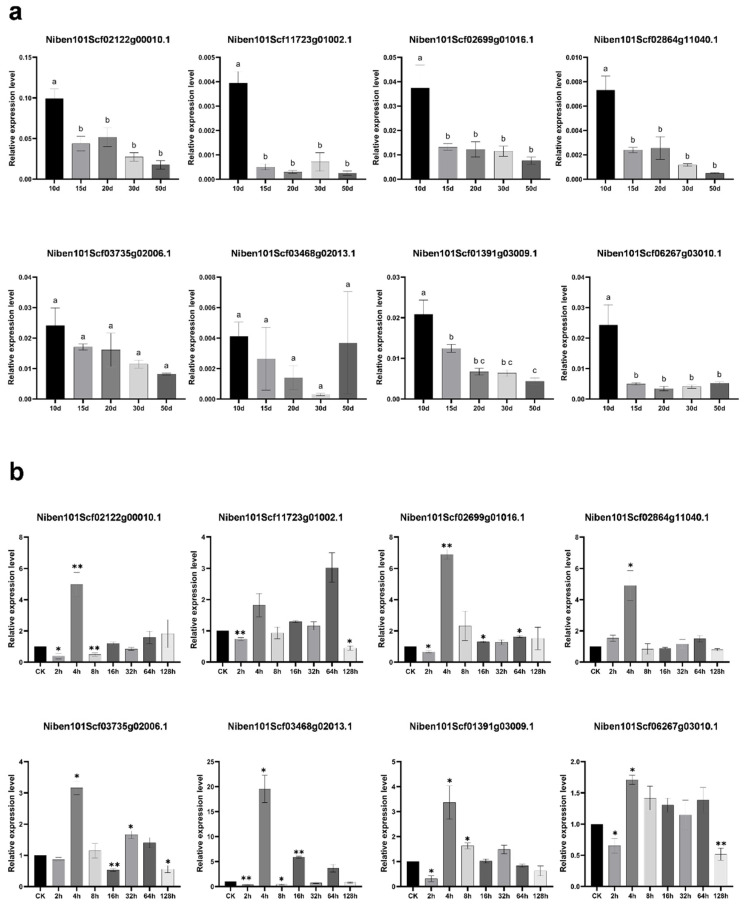
Expression patterns of m^6^A demethylase candidate genes in *N. benthamiana* analyzed by Reverse Transcription Quantitative Polymerase Chain Reaction (RT-qPCR). (**a**) Expression profiles of m^6^A demethylase candidate genes at different leaf developmental stages. Each value represents the mean ± SEM of three independent experiments. Statistical analysis was performed by one-way ANOVA, and different letters indicate significant differential expression; (**b**) Expression dynamics of m^6^A demethylase candidate genes at seven time points post *Potato virus Y* (PVY) infection. CK: healthy plants mock-inoculated with PBS. All RT-qPCR data were normalized with reference to the control samples. The values are expressed as the mean ± SEM (n = 3,* *p* < 0.05, ** *p* < 0.01).

The RT-qPCR at five leaf developmental stages and after PVY infection indicated that all eight candidate genes were expressed throughout the five leaf developmental stages, with significantly higher expression levels observed in primary leaves. As leaf development advanced, most of the genes showed a decreasing trend in expression. However, Niben101Scf03468g02013.1 (subgroup ALKBH9) displayed an increasing trend in expression during the later growth stages. Additionally, Niben101Scf02122g00010.1 (subgroup ALKBH9) consistently maintained relatively high expression levels across all developmental stages (Figure 2a). Furthermore, following PVY inoculation, the candidate genes exhibited differential expression patterns across the infection time course. Except for Niben101Scf11723g01002.1 (subgroup ALKBH10), which reached its highest transcript level at 64 h post-inoculation (hpi), the other genes demonstrated the most significant responses at 4 hpi. Notably, Niben101Scf03468g02013.1 (subgroup ALKBH9) was significantly upregulated (20-fold). In contrast, Niben101Scf06267g03010.1 (subgroup ALKBH9) exhibited relatively minor fluctuations and a lower overall response level during the viral infection period. These results indicate these candidate genes influence the response to PVY infection (Figure 2b). Given that Niben101Scf02122g00010.1 (subgroup ALKBH9) exhibited higher expression levels than other genes across various leaf developmental stages and showed a pronounced response to PVY infection, it was selected and designated NbALKBH1L for subsequent functional characterization.

### 2.3. Construction of Mutant Vectors and Corresponding Phenotypic Observation

**Figure 3:**
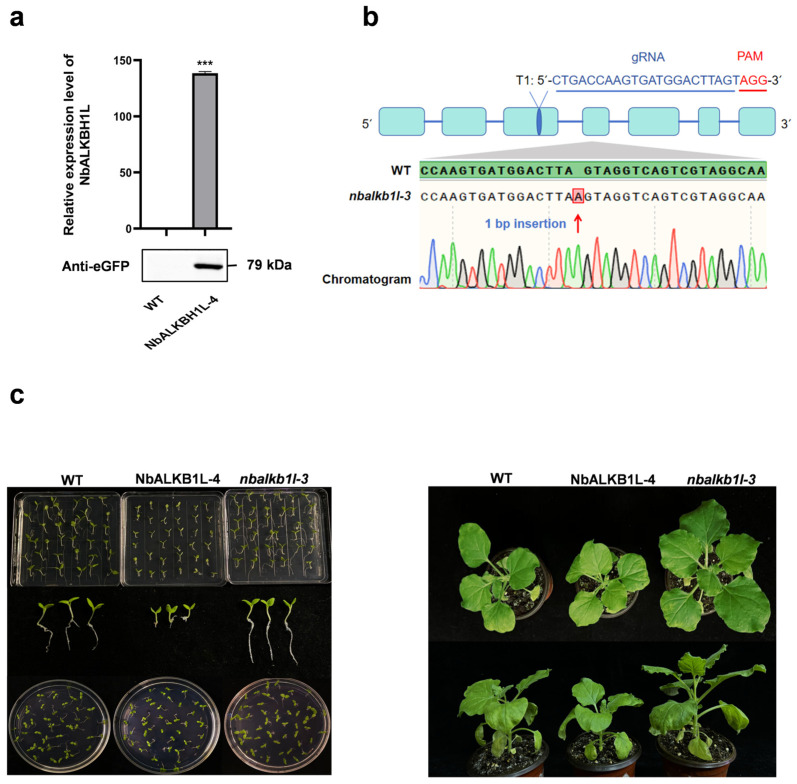
Construction of NbALKBH1L mutants. (**a**) RT-qPCR and Western blot analyses confirming effective translation and expression of NbALKBH1L in mutants. The values are expressed as the mean ± SEM (two-sided *t*-test, n = 3; *** *p* < 0.001); (**b**) CRISPR/Cas9-mediated genotyping of the *nbalkbh1l-3* mutant. The diagram illustrates single-guide RNAs (sgRNAs) designed to specifically target exons of NbALKBH1L. Red letters indicate the protospacer adjacent motif (PAM). Second-generation transgenic plants were genotyped by sequencing the genomic regions flanking the target site. Red arrows indicate the editing sites. The *nbalkbh1l-3* mutant carries a homozygous 1-bp insertion; (**c**) (**Left**): Seedling emergence of NbALKBH1L overexpression mutants and gene-edited mutants compared to Wild Type (WT) *N. benthamiana*. (**Right**): Growth phenotypes of *N. benthamiana* mutant plants.

We obtained NbALKBH1L-overexpressing mutant plants using the *Agrobacterium*-mediated transformation system in *N. benthamiana*. Western blot analysis with a GFP antibody and RT-qPCR confirmed significantly elevated NbALKBH1L expression levels in the NbALKBH1L-4 line (Figure 3a). Concurrently, we successfully generated gene-edited mutants of NbALKBH1L using CRISPR/Cas9 technology by introducing a single-base insertion near the predicted target site (Figure 3b). Homozygous screening subsequently yielded the stably inherited *nbalkbh1l-3* knockout line. Morphological observations of the mutant plants revealed that during germination, the knockout mutants exhibited a shorter germination time compared to both the wild-type (WT) and overexpressing mutants, while the overexpressing mutants required a longer germination time than the WT *N. benthamiana*. Further hydroponic culture experiments demonstrated that the knockout of NbALKBH1L enhanced the root development capacity of *N. benthamiana* compared to the WT. In contrast, the overexpressing mutants exhibited slower root growth (Figure 3c).

### 2.4. NbALKBH1L Possesses the Ability to Demethylate m^6^A Methylation Modifications

**Figure 4:**
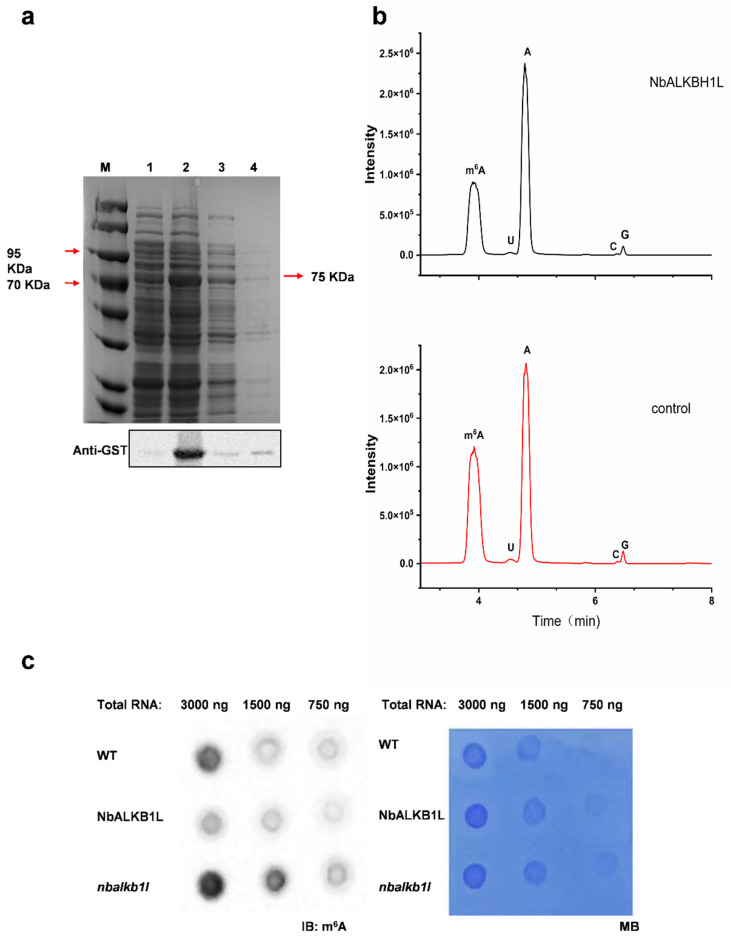
Validating NbALKBH1L enzymatic activity in vivo and in vitro. (**a**) Soluble expression of NbALKBH1L recombinant protein induced by 0.1 mM IPTG and Western blot analysis. M: Protein molecular weight marker; Lane 1: Uninduced control (0 h); Lane 2: Sample induced with 0.1 mM IPTG for 6 h; Lane 3: Total bacterial lysate; Lane 4: Supernatant after lysis; (**b**) The m^6^A demethylase activity of the purified NbALKBH1L protein, expressed prokaryotically, was analyzed using liquid chromatography–mass spectrometry (LC-MS); (**c**) Detection of m^6^A levels in total RNA from NbALKBH1L mutants and Wild Type *N. benthamiana*. (**Left**): Dot Blot analysis of total RNA; (**Right**): Methylene blue staining confirming equal loading.

To investigate whether NbALKBH1L possesses m^6^A demethylase activity, we expressed the full-length NbALKBH1L as a GST-tagged fusion protein in *Escherichia coli* and purified it (Figure 4a). An in vitro demethylation assay was then performed using synthesized m^6^A-modified single-stranded RNA as the substrate. Liquid chromatography-tandem mass spectrometry (LC-MS/MS) analysis of the digested nucleotides revealed that NbALKBH1L removed the m^6^A modifications from the test RNA compared to the control group (Figure 4b), demonstrating its ability to eliminate m^6^A methylation from single-stranded RNA in vitro. Furthermore, dot blot analysis showed that overexpression of NbALKBH1L reduced the m^6^A level in *N. benthamiana*, whereas knocking out NbALKBH1L increased the m^6^A level (Figure 4c). Collectively, these findings indicate that NbALKBH1L functions as an m6A demethylase in *N. benthamiana.*

### 2.5. NbALKBH1L Positively Regulates PVY Infection

**Figure 5:**
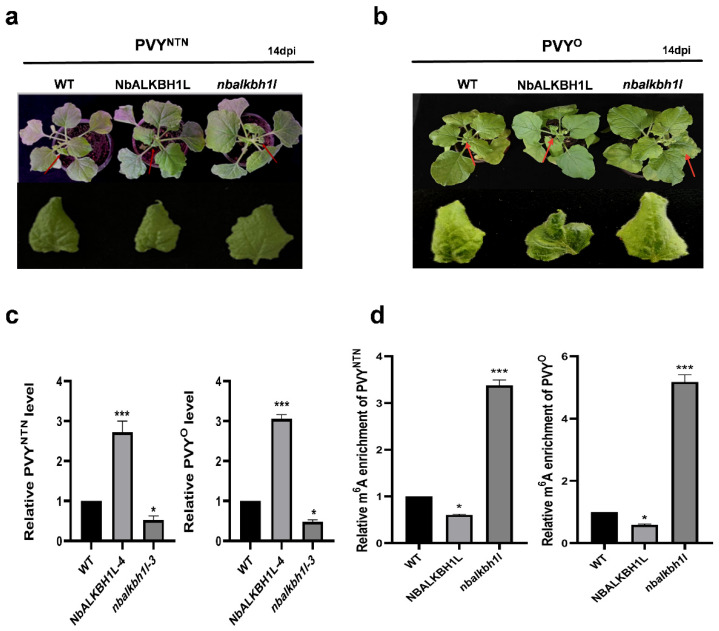
NbALKBH1L regulates PVY^NTN^ infection via m^6^A modification. (**a**) Phenotypes of overexpression mutants (NbALKBH1L-OE) and gene-edited knockout mutants (*nbalkbh1l*) at 14 dpi with PVY^NTN^, compared to Wild Type *N. benthamiana*; (**b**) Phenotypic comparison of mutants inoculated with PVY^O^ at 14 dpi, with WT as control; (**c**) RT-qPCR analysis of viral RNA accumulation in mutants infected with PVY^NTN^ or PVY^O^ at 14 dpi. The values are expressed as the mean ± SEM (two-sided *t*-test, n = 3, * *p* < 0.05, *** *p* < 0.001); (**d**) m^6^A enrichment levels in PVY genomic RNAs detected by m^6^A immunoprecipitation-qPCR (m^6^A-IP-qPCR) in NbALKBH1L mutants and WT plants infected with PVY^NTN^ or PVY^O^. The values are expressed as the mean ± SEM (two-sided *t*-test, n = 3, * *p* < 0.05, *** *p* < 0.001).

To investigate whether NbALKBH1L broadly regulates PVY infection, we inoculated plants with two PVY strains, PVY^NTN^ and PVY^O^. At 14 days post-inoculation (dpi), there was no significant difference in symptom presentation between the two strains. However, plants inoculated with either strain developed typical PVY symptoms, including mosaic patterns and leaf deformation. In contrast, the overexpression mutants exhibited more severe leaf deformation, while the knockout mutants showed attenuated symptoms compared to the WT (Figure 5a,b). Subsequently, RNA was extracted from the symptomatic new leaves of inoculated plants to assess viral load at both the transcriptional levels. The results revealed that, compared to WT plants, the overexpression mutant exhibited significantly elevated levels of PVY RNA. Conversely, the knockout mutant showed reduced accumulation of PVY RNA (Figure 5c). Furthermore, we quantified m^6^A levels on PVY genomic RNA in PVY-infected mutant and WT plants using m^6^A immunoprecipitation followed by qPCR (m^6^A-IP-qPCR) (Figure 5d). This analysis demonstrated lower m^6^A abundance on PVY RNA in the overexpression mutant compared to WT, while higher m^6^A abundance was detected on PVY RNA in the knockout mutant. These findings suggest that NbALKBH1L modulates PVY infection, likely by regulating m^6^A abundance on the PVY genome.

### 2.6. PVY CI Interacts with NbALKH1L

**Figure 6:**
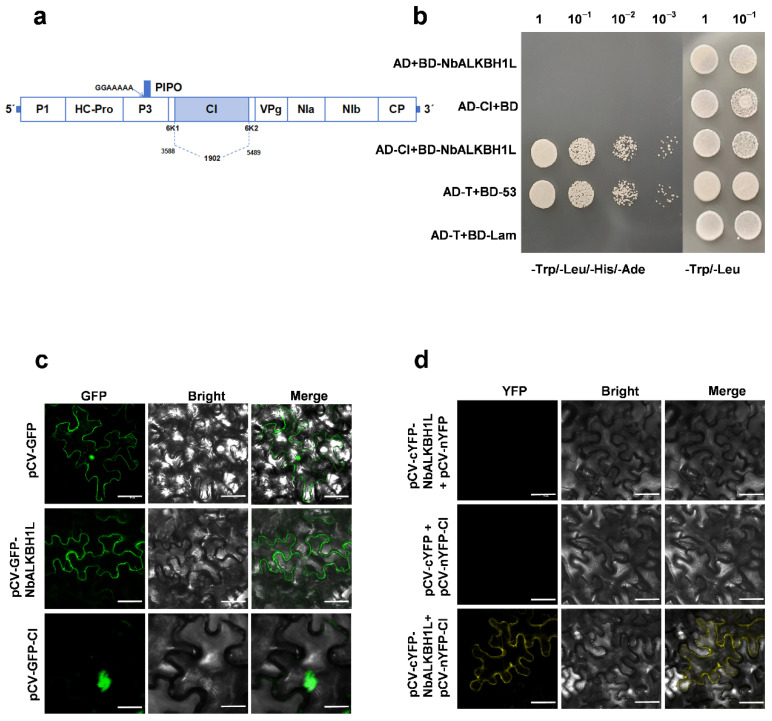
Interaction between NbALKBH1L and PVY CI. (**a**) Schematic representation of PVY genomic RNA organization; (**b**) The interaction between NbALKH1L and the PVY CI protein was confirmed using a Yeast two-hybrid (Y2H) assay. Yeast co-transformed with AD-T and BD-53 served as the positive control, while yeast co-transformed with AD-T and BD-Lam served as the negative control; (**c**) Subcellular localization of NbALKBH1L-GFP and CI-GFP fusion proteins observed by confocal laser scanning microscopy (CLSM). Empty GFP vector served as control. Scale bars: 50 μm; (**d**) The interaction between NbALKH1L and the PVY CI protein was validated using Bimolecular fluorescence complementation (BiFC). NbALKBH1L and CI were fused to N-terminal (YN) and C-terminal (YC) fragments of yellow fluorescent protein (YFP), respectively. Fluorescence reconstitution indicates protein–protein interaction. Scale bars: 50 μm.

Initial screening using Yeast two-hybrid (Y2H) assays was performed to investigate potential interactions between NbALKH1L and the eleven proteins encoded by PVY (Figure 6a). This analysis revealed a specific interaction between NbALKH1L and the PVY CI (Figure 6b). To further validate this interaction, subcellular localization studies and Bimolecular fluorescence complementation (BiFC) assays were conducted. The results demonstrated that both NbALKH1L and CI localize to the cytoplasm. Importantly, the BiFC assays confirmed a direct interaction between NbALKH1L and CI within this cellular compartment (Figure 6c,d). Collectively, these findings suggest that PVY may exploit the interaction between its CI protein and the host protein NbALKH1L to recruit the latter for involvement in regulating viral infection.

### 2.7. Integrated Analysis of IP-MS and RNA-Seq

**Figure 7:**
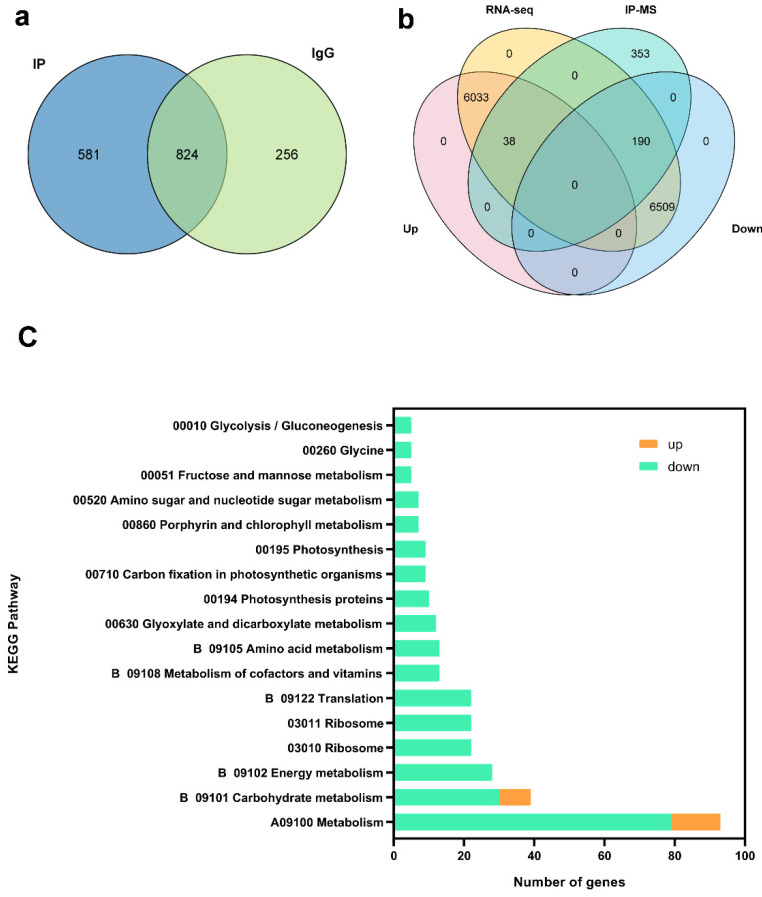
Integrated analysis of NbALKBH1L interactome (IP-MS) and transcriptome (RNA-seq) in *N. benthamiana*. (**a**) Venn diagram of proteins identified in anti-GFP immunoprecipitation (IP) versus IgG control. The intersection represents non-specifically bound proteins, yielding 581 high-confidence NbALKBH1L-interacting proteins after background subtraction; (**b**) Integrated analysis of IP-MS and RNA-seq data. Virus-responsive genes were categorized as upregulated (up) or downregulated (down), identifying 38 upregulated and 190 downregulated proteins that interact with NbALKBH1L; (**c**) KEGG pathway enrichment analysis of the filtered interacting proteins. *X*-axis: Gene count; *Y*-axis: Pathway names.

To further investigate the potential mechanism by which NbALKBH1L regulates viral infection, we identified 581 NbALKBH1L-interacting proteins through IP-MS (Figure 7a). Subsequently, RNA-seq analysis revealed 12,770 differentially expressed genes (DEGs) in response to viral infection; 6071 upregulated and 6699 downregulated genes. An integrated analysis of these datasets identified 38 genes that were upregulated during viral infection. KEGG pathway enrichment analysis indicated significant enrichment of the studied genes in carbohydrate metabolism and general metabolic pathways, highlighting their biological relevance. Additionally, 190 genes were downregulated post-infection, with KEGG analysis revealing enrichment in metabolic pathways, carbohydrate metabolism, energy metabolism, ribosome biogenesis, and translation (Figure 7b,c). Notably, within the metabolic pathways, we identified key disease resistance-related genes, including nucleoside diphosphate kinase (NDPK) [41], cytochrome P450s [42], ascorbate peroxidase (APX) [43], and linoleate 9S-lipoxygenase (9S-LOX) [44]. Beyond these enriched pathways, we also identified several genes with established roles in regulating viral infections, such as poly (A)-binding protein (PABP) [45], ribosomal proteins (RPs) [46,47], remorin proteins [48], serine carboxypeptidase (SCP) [49], subtilisin-like protease (SLP) [50], protein phosphatase 2C family protein (PP2C) [51], aldo-keto reductase family member (AKR) [52], and plastid-lipid associated protein (PAP)/fibrillin family protein (FBN) [53].

## 3. Discussion

m^6^A modification is a dynamic, reversible biological process. The obesity-associated protein FTO, the first identified m^6^A demethylase in animals, catalyzes demethylation by sequentially oxidizing m^6^A to unstable intermediates, hm^6^A and f^6^A, followed by the removal of one molecule of formaldehyde and formic acid, respectively, ultimately reverting to adenine (A) [54]. Another well-characterized m^6^A demethylase in mammals is ALKBH5. Both FTO and ALKBH5 belong to the α-ketoglutarate and Fe(II)-dependent dioxygenase family. However, unlike FTO, ALKBH5 directly catalyzes m^6^A demethylation without generating detectable intermediates [55]. To date, no FTO homologs have been identified in plants. The known plant m^6^A demethylases are all homologs of ALKBH5 and contain the conserved AlkB catalytic domain. In this study, we conducted a bioinformatic analysis to identify AlkB family genes in *N. benthamiana*. Subsequent expression profiling revealed that NbALKBH1L exhibited relatively high transcript abundance across various leaf developmental stages and showed significant responsiveness to PVY infection. Therefore, we selected NbALKBH1L as a candidate m^6^A demethylase gene for further investigation. Enzymatic assays subsequently confirmed that NbALKBH1L possesses m^6^A demethylase activity and is localized in the cytoplasm. These findings suggest that NbALKBH1L, a homolog of *A. thaliana* ALKBH9B, may have similar biological functions and could play an important role during PVY infection.

Multiple members of the ALKBHs have been reported to participate in the dynamic regulation of m^6^A modification, which influences RNA stability, translation efficiency, and gene expression. In *Arabidopsis*, ALKBH9B enhances the stability of viral RNA and negatively regulates plant antiviral immune responses by removing m^6^A modifications from the AMV genome [40]. In rice, ALKBH9 is involved in pollen development and male fertility by mediating the m^6^A demethylation of mRNAs that encode key regulators of tapetum programmed cell death, such as TDR and GAMYB [56]. In watermelon, the specific early upregulation of ClALKBH4B in resistant varieties upon *Cucumber green mottle mosaic virus* (CGMMV; *Tobamovirus viridimaculae*) infection indicates a critical role in antiviral immunity. The proposed mechanism suggests that ClALKBH4B reduces m^6^A methylation on viral RNA, thereby activating the plant’s defense response [57]. The tomato ALKBH2 protein has also been reported to possess m^6^A demethylase activity. SlALKBH2 increases the stability of SlDML2 mRNA by specifically removing the m^6^A modification from the DNA demethylase gene SlDML2, thereby promoting tomato fruit ripening [58]. In this study, we identified the m^6^A demethylase NbALKBH1L as a key regulator of *N. benthamiana* responses to PVY infection. Following PVY inoculation, viral accumulation was significantly lower in knockout mutants compared to WT plants, while accumulation was higher in NbALKBH1L-overexpressing lines. Additionally, PVY genomic RNA exhibited increased m^6^A abundance in the knockout mutants. The results strongly indicate that NbALKBH1L acts as a positive regulator of viral infection. Concurrently, phenotypic studies revealed that root development in NbALKBH1L functional knockout mutants was enhanced compared to that in wild-type and overexpressing mutants. Thus, NbALKBH1L not only actively participates in regulating PVY infection but may also play a role in controlling plant growth and development.

**Figure 8:**
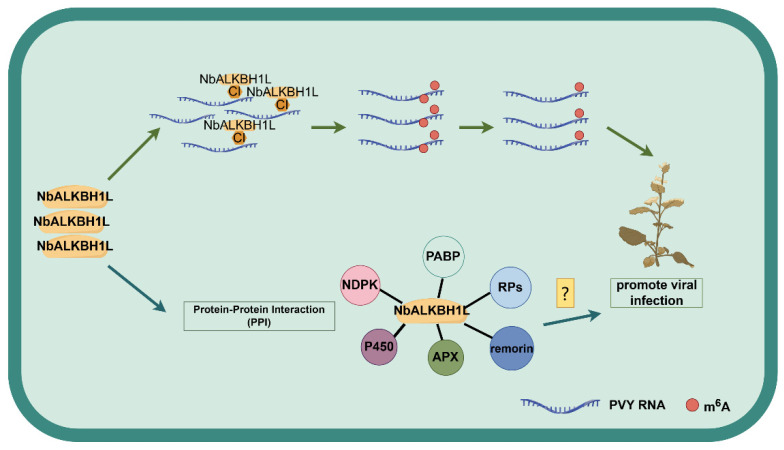
NbALKBH1L is involved in the putative regulatory mechanism of plant virus infection. During PVY infection, NbALKBH1L may function as a regulatory nexus. On one hand, the CI protein potentially recruits NbALKBH1L through protein–protein interactions, enhancing viral genome stability by reducing m^6^A modifications, which facilitates PVY infection. On the other hand, NbALKBH1L may also regulate the expression of pathway-related genes through m^6^A-mediated epigenetic modulation, thereby coordinating the host’s defense response. This diagram was created using FigDraw (https://www.figdraw.com/static/index.html, accessed on 21 July 2025).

Recent studies in epitranscriptomics have revealed that core components of m^6^A modification play crucial regulatory roles during viral infections by interacting with viral proteins [59,60]. For instance, the CP of AMV appears to recruit the host m^6^A demethylase through protein–protein interactions. This recruitment leads to a reduction in the abundance of m^6^A modifications on the AMV genomic RNA, thereby enhancing viral genomic stability and promoting AMV infection [40]. Similarly, the RNA-dependent RNA polymerase (RdRp) of *Pepino mosaic virus* (PepMV; *Potexvirus pepini*) interacts with HAKAI, a component of the plant m^6^A methyltransferase complex. This interaction activates the autophagy pathway, resulting in the degradation of HAKAI and the subsequent impairment of the m^6^A methyltransferase complex’s function. This mechanism likely protects the viral genome from m^6^A-mediated antiviral responses [61]. Furthermore, the replication enzyme NIb of *Wheat yellow mosaic virus* (WYMV; *Bymovirus triticitessellati*) induces the nuclear export of the wheat methyltransferase TaMTB. This relocalization facilitates m^6^A modification at specific sites on viral RNA1, which promotes the stability of the viral RNA [62]. Collectively, these examples illustrate the diverse strategies employed by plant viruses to manipulate the host m^6^A modification machinery for their own benefit. In this study, we observed that the PVY genome undergoes dynamic m^6^A modification. The viral-encoded CI protein interacts with the plant m^6^A demethylase NbALKBH1L, and both proteins localize to the cytoplasm—findings that are consistent with previous reports [40,63]. The CI protein is a key component of the viral replication complex (VRC) in *Potyviruses*, the genus that includes PVY. It plays essential roles in viral RNA replication, interplastid trafficking, and cell-to-cell movement [64,65,66]. Relevant studies suggest that CI proteins may interact with host factors to facilitate viral infection. For instance, the CI proteins of *Turnip mosaic virus* (TuMV; *Potyvirus rapae*) and *Soybean mosaic virus* (SMV; *Potyvirus glycitessellati*) interact with host dynamin-related proteins (DRPs), co-opting the host’s endocytic machinery to support viral replication and movement [67,68]. In this study, it remains to be further elucidated whether the CI protein recruits plant m^6^A demethylases through protein interactions, similar to the mechanisms described in the aforementioned studies, thereby reducing m^6^A modifications that enhance viral genome stability and facilitate PVY infection (Figure 8).

Integrated analysis of IP-MS and RNA-seq data identified over two hundred proteins interacting with NbALKBH1L in *N. benthamiana* that were significantly responsive to PVY infection. The enrichment analysis showed that these proteins were notably abundant in metabolic pathways. Importantly, proteins within these pathways, such as NDPK, cytochrome P450, and ascorbate peroxidase, have well-established roles in regulating pathogen infection, as documented in previous studies. For instance, NDPK expression is upregulated in plants upon pathogen challenge [41]. P450 enzymes constitute a large family of autoxidizable, heme-containing proteins classified as monooxygenases. Members of the P450 family (AtCYP76C2 and GmCYP82A3) play important roles in plant resistance to bacterial and fungal infections, respectively [42]. While ascorbate peroxidase OsAPX8 enhances rice resistance to bacterial blight by scavenging reactive oxygen species (ROS) and maintaining intracellular homeostasis [43]. Beyond the significantly enriched pathways, we identified proteins responsive to PVY infection that are implicated in regulating viral pathogenesis. These proteins include PABP, ribosomal proteins, and remorin. PABP plays a critical role in translational regulation during plant antiviral defense by facilitating the preferential translation of antiviral mRNAs through dynamic binding with eIF4G/eIFiso4G isoforms, thereby activating immune responses [45]. In *N. benthamiana*, reducing the expression of the ribosomal protein RPS6 influences the levels of CMV, TuMV, and *Potato virus A* (PVA; *Potyvirus atuberosi*). On the other hand, silencing certain members of the RPS6 gene family increases the plant’s resistance to *Tomato spotted wilt virus* (TSWV; *Orthotospovirus tomatomaculae*) and *Potato virus X* (PVX; *Potexvirus ecspotati*) [46,47]. Studies on remorin indicate that its accumulation negatively correlates with the cell-to-cell movement of PVX. Furthermore, remorin interacts with the PVX movement protein TGBp1, thereby impeding viral trafficking [48]. In the field of plant immunity, extensive research indicates that intricate and sophisticated molecular regulatory networks within plants are crucial for defending against viral infections. Compared to control groups, proteins interacting with NbALKBH1L exhibited significantly differential expression during PVY infection, suggesting its potential role as a pivotal regulatory hub. NbALKBH1L may recruit associated proteins through interactions, thereby playing an important role in the plant’s response to viral infections. Concurrently, studies have revealed that genes involved in sugar metabolism and signaling—such as α-L-fucosidase and glycosyltransferase family proteins—displayed significantly decreased levels of m^6^A modification, yet increased transcriptional levels following CGMMV infection. This suggests that m^6^A modifications may regulate plant metabolic processes by influencing the expression of these genes, consequently enhancing plant defense responses [57]. As an m^6^A demethylase, NbALKBH1L functions, at least partially, by recruiting interacting partners. However, whether it also coordinates host defense responses by epigenetically regulating the expression of pathway-related genes via m^6^A modification remains to be elucidated (Figure 8).

## 4. Materials and Methods

### 4.1. Virus Inoculation and Sampling

Leaf samples of *N. benthamiana* were collected on days 10, 15, 20, 25, and 50 post-sowing to analyze the expression patterns of candidate genes across different developmental stages. Additionally, at the 8-leaf stage (one month after transplanting), the plants were inoculated with the PVY, while the control group was treated with phosphate-buffered solution. Samples were collected at 0, 2, 4, 8, 16, 32, 64, and 128 hpi after PVY inoculation to elucidate the expression patterns of candidate genes under PVY infection. Mutant plants, including NbALKBH1L overexpression lines and *nbalkbh1l-Cas9* knockout mutants, were cultivated under the same conditions as the WT. Inoculation was conducted at the same developmental stage, with sampling 10–14 dpi, once symptoms had emerged. For this experiment, leaf samples were collected from newly emerging leaves and from leaves at the onset of symptoms. Sample preservation adhered to the same protocol [69].

### 4.2. Identification of AlkB Homolog (ALKB) Gene Family Members in N. benthamiana

A BLASTP analysis [70] was conducted on the *N. benthamiana* genome using the known AlkB-encoding protein sequence from *A. thaliana* as a reference. TBtools (v1.09876), with default settings, was employed to identify potential AlkB-encoding proteins. Redundant sequences were filtered using NCBI (https://www.ncbi.nlm.nih.gov/), followed by structural domain validation with CD-Search (https://www.ncbi.nlm.nih.gov/Structure/bwrpsb/bwrpsb.cgi, accessed on 13 June 2023) [71], which excluded sequences lacking the conserved AlkB domain. Finally, a multiple sequence alignment of the confirmed AlkB domain-containing proteins from *N. benthamiana*, along with known AlkB family members from *A. thaliana* and Homo sapiens, was performed using ClustalW in MEGA 7.0 with default parameters [72]. The aligned sequences were then used to construct a phylogenetic tree to elucidate evolutionary relationships.

### 4.3. Analysis of the Domain Structure and Physicochemical Properties of the m^6^A Demethylase Gene in N. benthamiana

A multiple sequence alignment and phylogenetic analysis of the complete sequences of eight candidate genes were conducted using the ClustalW tool in MEGA 7.0 software with standard settings. An analysis of conserved domains in the eight candidates were examined using Batch CD-Search, and the resulting files (in .txt format) were later visualized with TBtools. Furthermore, the molecular weight (MW), isoelectric point (pI), and subcellular localization of these proteins were predicted using the online tools ExPASy (https://web.expasy.org/compute_pi/, 26 June 2023) and Cell-PLoc (http://www.csbio.sjtu.edu.cn/bioinf/Cell-PLoc-2/, 28 June 2023) [73].

### 4.4. Total RNA Extraction and RT-qPCR Analysis

Total RNA was extracted from the samples using a Total RNA extraction reagent (Biosharp, Beijing, China), followed by reverse transcription with the RT SuperMix for qPCR kit (Vazyme, Nanjing, China). Quantitative assessment was performed via real-time fluorescent quantitative PCR (RT-qPCR) utilizing the CFX Duet system from Bio-Rad (Hercules, CA, USA). The NtUBI transcript was used as an internal control for normalization. Relative expression levels were calculated using the 2^−∆∆CT^ method [74] and normalized to the expression levels in mock-inoculated control plant tissues to analyze post-PVY infection expression patterns. The final data were visualized using GraphPad Prism 10 software.

### 4.5. Plasmid Construction

For vector construction, Gibson assembly was utilized to generate: Prokaryotic Expression Vector pGEX, Overexpression vector pART27-eGFP, Y2H Vectors pGADT7/pGBKT7, and Subcellular Localization Vector pCV-GFP. Purified restriction digests of these vectors were ligated to target gene fragments using the cloning kit from Vazyme (ClonExpress II One Step Cloning Kit), China. In contrast, traditional restriction-ligation cloning was employed for: BiFC vectors, pCV-nYFP-C and pCV-cYFP-N, CRISPR-Cas9 Knockout Vector pHSbdcaa9i-tRNA(k5). Specifically, plasmid backbones and insert fragments were digested and followed by ligation with T4 ligase (New England Biolabs, Ipswich, MA, USA).

### 4.6. Protein Expression and Purification

The recombinant NbALKBH1L construct ligated into pGEX-6p-1 was introduced into Rosetta-gami 2(DE3) competent cells for protein production. The expression was triggered with 0.1 mM IPTG at 16 °C for 6 h. After harvesting, the cells were resuspended in PBS (pH 7.4) and lysed using an ultrasonic disruptor (Ningbo Scientz Biotechnology Co., Ltd., Ningbo, China). The mixture was then centrifuged, and the supernatant was subjected to affinity chromatography purification with proteinIso™ GST Resin (TRANS, Beijing, China). The target protein was eluted using reduced glutathione and the fractions were analyzed using SDS-PAGE.

### 4.7. Liquid Chromatography-Tandem Mass Spectrometry

To assess the m^6^A demethylase activity of NbALKBH1L, an in vitro enzymatic assay was conducted using the purified recombinant protein GST:NbALKBH1L, as described in Section 4.6. The reaction mixture included 1 µg of synthetic m^6^A-modified single-stranded RNA substrate, the recombinant protein, and a control consisting of 10 mM reduced glutathione. The reaction was performed in a buffer containing 50 mM HEPES, 2 mM ascorbic acid (pH 7.0), 300 µM ammonium iron(II) sulfate hexahydrate, 500 µM α-ketoglutarate, and 300 U/mL RNase inhibitor at 25 °C for 6 h. The reaction was terminated by adding 5 mM EDTA and incubating at 95 °C for 10 min in a PCR machine. RNA was extracted from the reaction products using the phenol-chloroform-isoamyl alcohol (25:24:1) method. The purified single-stranded RNA (ssRNA) was denatured by heating at 95 °C for 10 min, followed by immediate cooling on ice for 2 min. To this mixture, 2 µL of 10× S1 nuclease buffer, 2 µL of 500 mM NaCl, and 2 µL of S1 nuclease were added, followed by incubation in a 37 °C water bath for 6 h. Subsequently, 10 µL of 10× alkaline phosphatase buffer, 10 µL of dilution buffer, 6 µL of alkaline phosphatase, and 54 µL of sterile water were added, and incubation continued at 37 °C for an additional 6 h [24,40,58]. The final product was diluted with 900 µL of sterile ddH_2_O and transferred to liquid chromatography (LC) injection vials. Qualitative and quantitative analyses of the samples were performed using an AB SCIEX QTRAP 4500 LC-MS/MS system (AB Sciex LLC, Framingham, MA, USA). The corresponding m^6^A signal intensity was subsequently analyzed.

### 4.8. Plant Transformation

Sterilized seeds were sown on a germination medium and cultured under controlled conditions for 4–5 weeks. Aseptic leaves were excised into small segments using a scalpel and placed onto pre-culture medium. *Agrobacterium tumefaciens* strains containing target constructs were suspended in liquid culture, harvested by centrifugation, and resuspended to an OD_600_ of 0.2. Leaves from 2–3-day pre-cultured *N. benthamiana* plants were immersed in the *Agrobacterium* suspension for 10–15 min. After co-cultivation, the leaf explants were blotted dry on sterile filter paper and transferred onto co-cultivation medium. All samples were maintained in darkness at 22 °C for 48–72 h. After approximately two days of co-cultivation, leaf segments were moved to a medium designed for callus induction. Calli that appeared within ten days were selected based on their morphological characteristics, such as a healthy and compact look, and then placed onto a selection medium that contained the necessary antibiotics. The cultures were kept at a temperature of 23 ± 2 °C for a period of 15 to 30 days. During the secondary selection, actively growing calli that were likely transgenic were then transferred to a shoot regeneration medium, with 4–5 calli per plate, and cultured under a light cycle of 16 h of light and 8 h of darkness for another 15 to 30 days. As the shoots developed, they were cut and moved to a rooting medium for 7 to 10 days to encourage strong growth.

### 4.9. Screening and Identification of Transgenic Plants

To create homozygous transgenic lines that overexpress the target gene, total RNA was extracted from T1-generation overexpression mutants and WT *N. benthamiana*. The expression levels of the gene were then assessed using RT-qPCR to identify plants with elevated target gene expression. The chosen plants were subcultured on 1/2 MS resistance medium and self-pollinated repeatedly until a stable segregation ratio was achieved, confirming the establishment of homozygous mutants. For the homozygous gene-edited knockout mutants, genomic DNA was extracted from T1-generation CRISPR-edited mutants and wild-type plants. The DNA extraction method was performed according to this protocol [75]. PCR amplification was performed with primers flanking the target site (approximately 150 bp upstream and downstream), and the resulting PCR products were sequenced. The sequencing data were analyzed using software DSDecodeM (http://skl.scau.edu.cn/dsdecode/, accessed on 27 December 2023) to pinpoint successfully edited plants. The edited lines were then subcultured on 1/2 MS resistance medium, self-pollinated until no segregation was observed, and verified as homozygous mutants through sequencing. The primers utilized in these experiments are detailed in Table S3.

### 4.10. Western Blot

Total protein was extracted from the test plants using a protein lysis buffer. Purified proteins or extracted plant proteins were separated by SDS-PAGE and subsequently electrotransferred onto a nitrocellulose membrane (Boster, Wuhan, China). The membrane was incubated with either an anti-GST antibody (1:1500, Zhongshan Golden Bridge, Beijing, China) or a GFP antibody (1:30,000, Sangon Biotech, Shanghai, China). Horseradish peroxidase (HRP)-conjugated secondary antibodies were selected based on the primary antibodies for immunoblotting detection. Finally, the membrane was developed using an imaging system (Peiqing Technology Co., Ltd., Shanghai, China).

### 4.11. Dot Blot

Total RNA was extracted and sample concentrations were diluted to 1500 ng/μL, 750 ng/μL, and 375 ng/μL. Samples were denatured and then rapidly cooled on ice. For each sample, 2 μL was sequentially spotted onto a NC membrane, air-dried at room temperature for 5 min, transferred to a 37 °C incubator for cross-linking for 1 h, and then washed with TBST for 5 min. After blocking, the diluted m^6^A antibody (1:5000, SySy, Gottingen, Germany) working solution was added, and the membrane was incubated at 4 °C overnight. Following this, the membrane was incubated with secondary antibody at room temperature for 2 h, developed using an imaging system, and finally stained with 0.2% methylene blue staining solution (Solarbio, Beijing, China).

### 4.12. Agrobacterium Infiltration

The PVY infectious clone was transformed into *A. tumefaciens* and cultured in a shaking incubator. When the bacterial culture reached OD_600_ ≈ 0.6, cells were harvested and resuspended in infiltration buffer (100 μL of 100 mM AS, 1 mL of 500 mM MES, and 1 mL of 500 mM MgCl_2_ were added, and the final volume was adjusted to 100 mL with ddH_2_O) to OD_600_ = 1.0. After 3 h of incubation at room temperature, the resuspended culture was infiltrated into the leaves of healthy WT *N. benthamiana* plants, as well as overexpression and knockout mutants, using a 1 mL syringe. The infiltrated plants were then placed in darkness for 24 h before being transferred to a growth chamber under standard growth conditions.

### 4.13. Yeast Two-Hybrid Assay

The pGBDT7-NbALKBH1L plasmid and the eleven proteins encoded by the PVY were co-transformed along with the pGADT7 recombinant plasmid. Colonies of yeast (co-transformants) that grew on the SD/-Trp-Leu plates were selected, diluted serially, and streaked onto quadruple dropout media. Colony growth was monitored after further incubation at 30 °C for 4–5 days.

### 4.14. BiFC and Subcellular Localization

The recombinant plasmids pCV-nYFP-CI, pCV-cYFP-NbALKBH1L, pCV-GFP-CI, and pCV-GFP-NbALKBH1L were transformed into *Agrobacterium* (strain GV3101), and the resuspension was prepared according to the method described in Section 4.12, followed by standing at room temperature for 3 h. For BiFC, the resuspensions of the two *Agrobacterium* strains were mixed in a 1:1 ratio and injected into tobacco leaves. The infiltration method for subcellular localization was consistent with the *Agrobacterium* infiltration method. After 48–72 h of infiltration, a 1–2 square centimeter leaf area near the infection site was prepared as a slide and observed for fluorescence under a laser confocal inverted microscope (Olympus FV3000, Tokyo, Japan).

### 4.15. m^6^A-IP-qPCR

m^6^A-IP-qPCR was conducted as previously described, with some modifications [70,71,76,77]. Total RNA (150 μg) was extracted from WT, overexpression, and knockout plants four weeks after inoculation (4 wpi) with PVY.

Total RNA was purified by DNase I treatment to eliminate DNA. Following this, the RNA was precipitated and purified using glycogen (Beyotime, Shanghai, China) and isopropanol. The RNA was divided into three portions: input sample, IP group and the IgG group. For the m^6^A IP group and the IgG group, 4 μg of anti-m^6^A polyclonal antibody (SySy, Gottingen, Germany) and anti-IgG antibody were added, respectively, and incubated on a vertical mixer at 4 °C for 4–6 h. Subsequently, 40 μL of equilibrated Protein A/G magnetic beads (Thermo Fisher Scientific, Waltham, MA, USA) were used for immunoprecipitation of each mixture by incubating at 4 °C for at least 6 h. The RNA reaction mixtures were thoroughly washed, and the RNA was recovered for subsequent RT-qPCR analysis.

### 4.16. Immunoprecipitation–Mass Spectrometry

Plant proteins were extracted from PVY-inoculated overexpressing plants using a protein lysis buffer that contained a protease inhibitor cocktail. The cell lysate was split and then added to IgG magnetic beads and GFP magnetic beads that had been pre-treated with antibodies, respectively.

After incubation at room temperature for 2 h, the supernatants were discarded, and the beads were washed twice. Subsequently, the beads were resuspended and subjected to a boiling water bath for 10 min. Following centrifugation, the supernatants were collected for Western blot analysis. Silver staining was then performed to verify the integrity of the cellular proteins, and differential protein bands between the IgG and GFP-IP groups were excised for mass spectrometry (MS) identification. Bioinformatics analysis was performed on the raw MS data to interpret the results.

### 4.17. RNA-Seq

Total plant RNA was extracted and the sequencing libraries were constructed by Biomarker Technologies Co., Ltd. (Beijing, China). The sequencing was performed on an Illumina NovaSeq platform (Illumina, Inc., San Diego, CA, USA) according to the manufacturer’s instructions. Raw sequencing data were processed and analyzed using the bioinformatics platform BMKCloud (www.biocloud.net).

## 5. Conclusions

In summary, we identified NbALKBH1L as an m^6^A demethylase in *N. benthamiana* and demonstrated its influence on viral infection by modulating the m^6^A abundance on the PVY genomic RNA. Furthermore, by screening for interactions between NbALKBH1L and viral proteins, combined with IP-MS and RNA-seq analyses, we constructed an interaction network centered on NbALKBH1L. This integrative approach revealed the complexity of the regulatory processes by which NbALKBH1L positively regulates PVY infection. Our findings not only enhance the understanding of plant m^6^A demethylases but also establish a role for NbALKBH1L in regulating the growth and development of *N. benthamiana*. Collectively, this work provides a foundational dataset for deciphering the regulatory mechanisms of m^6^A modification in the plant “growth-defense trade-off”.

## Data Availability

The Solanaceae gene sequences analyzed in this study are publicly available in the Sol Genomics Network (SGN). The raw sequence data for this experimental result has been deposited in the NCBI Sequence Read Archive (SRA) database (accession number: PRJNA1308269).

## Supplementary Materials

The following supporting information can be downloaded at: https://www.mdpi.com/article/10.3390/plants14243796/s1, Table S1: Basic information of *Nicotiana benthamiana* m^6^A demethylase candidate genes; Table S2: Analysis of the conserved structural domains of the AlkB family members of *N. benthamiana* IP-MS/RNA-seq Analysis; Table S3: Primer sequences used in this experiment. Figure S1: Analysis of the conserved structural domains of the AlkB family members of *Nicotiana benthamiana*; Figure S2: Screening of NbALKBH1L overexpression Mutants; Figure S3: The Y2H assay was performed to validate the interactions between NbALKH1L and 11 proteins encoded by PVY; Supplementary Data: IP-MS screening results.
