# Supplementary material for: ALKBH1L Is an m6A Demethylase and Mediates PVY Infection in Nicotiana benthamiana Through m6A Modification
Source: Plants (Basel). 2025 Dec 13;14(24):3796. doi: 10.3390/plants14243796 (PMC12736721; doi:10.3390/plants14243796)
Supplement: Supplementary file 1 [file plants-14-03796-s001.zip › Table S1.pdf]

Table S1 Basic information of *Nicotiana benthamiana* m^{6}A demethylase candidate genes

**Protein family**	**Gene ID**	**Protein Length （aa）**	**Molecular Weight（KD）**	**Theoretical pI**	**subcellular localization**
AlkB	Niben101Scf03735g02006.1	540	60593.19	5.61	Nucleus
	Niben101Scf11723g01002.1	540	59663.98	6.06	Chloroplast
	Niben101Scf02699g01016.1	296	32617.68	9.34	Nucleus Chloroplast
	Niben101Scf02122g00010.1	457	51929.95	8.53	Chloroplast
	Niben101Scf02864g11040.1	192	22015.31	9.12	Nucleus
	Niben101Scf03468g02013.1	540	61855.06	6.51	Chloroplast Nucleus
	Niben101Scf06267g03010.1	537	60199.84	6.10	Nucleus
	Niben101Scf01391g03009.1	296	32543.51	9.37	Chloroplast
