# Supplementary material for: ALKBH1L Is an m6A Demethylase and Mediates PVY Infection in Nicotiana benthamiana Through m6A Modification
Source: Plants (Basel). 2025 Dec 13;14(24):3796. doi: 10.3390/plants14243796 (PMC12736721; doi:10.3390/plants14243796)
Supplement: Supplementary file 1 [file plants-14-03796-s001.zip › Table S2.pdf]

Table S2: Summary of disease resistance-related genes from integrated IP-MS/RNA-Seq analysis

ID	Description	References
Niben101Scf04328g00019.1	nucleoside diphosphate kinase 2 LENGTH=231	(Cao et al. 2008)
Niben101Scf03734g00002.1	Cytochrome P450 superfamily protein LENGTH=539	(Chakraborty et al. 2023)
Niben101Scf06195g00002.1	ascorbate peroxidase 1 LENGTH=250	(Gong et al. 2024)
Niben101Scf02367g05038.1	ascorbate peroxidase 4 LENGTH=349	
Niben101Scf02688g02014.1	Linoleate 9S-lipoxygenase 6	(An et al. 2019)
Niben101Scf10464g00005.1	Polyadenylate-binding protein 4	(Wang et al. 2022)
Niben101Scf06437g01008.1	50S ribosomal protein L13	(Rajamäki et al. 2017; Helderman et al. 2022)
Niben101Scf10885g00015.1	50S ribosomal protein L22	
Niben101Scf06436g02010.1	Remorin	(Raffaele et al. 2009)
Niben101Scf05890g02009.1	Serine carboxypeptidase-like 48	(Liu et al. 2008)
Niben101Scf02358g02002.1	Serine carboxypeptidase-like 27	
Niben101Scf05325g00014.1	Serine carboxypeptidase 24	
Niben101Scf12205g02003.1	Subtilisin-like protease	(Figueiredo et al. 2018)
Niben101Scf19534g00001.1	Protein phosphatase 2C family protein LENGTH=298	(Hu et al. 2009)
Niben101Scf00698g02019.1	Aldo-keto reductase family 4 member	(Chen et al. 2023)
Niben101Scf06661g00007.1	Plastid-lipid associated protein PAP / fibrillin family protein LENGTH=299	(Sappah et al. 2024)
