# Supplementary material for: ALKBH1L Is an m6A Demethylase and Mediates PVY Infection in Nicotiana benthamiana Through m6A Modification
Source: Plants (Basel). 2025 Dec 13;14(24):3796. doi: 10.3390/plants14243796 (PMC12736721; doi:10.3390/plants14243796)
Supplement: Supplementary file 1 [file plants-14-03796-s001.zip › Table S3.pdf]

Table S3: Primer sequences used in this experiment

Application	Primer Name	Sequence (5'-3')
Expression patterns of m^{6}A demethylase candidate genes	q-NtUBI-F	TCCAGGACAAGGAGGGTATCC
	q-NtUBI-R	GTCAGCCAAGGTCCTTCCATCC
	q-0010.1-F	TTTCATGGCTATGTTAAGGAATCTG
	q-0010.1-R	GACATTTCATCTAACCTTCTCTGATG
	q-1002.1-F	CCTGGTGTCCCAACACAATA
	q-1002.1-R	GGACGAATAGGAGGTAGCATAAC
	q-1016.1-F	GGAGTATTCACAGCCTTTCCT
	q-1016.1-R	TTCCCTTCACTGTCGCTTAC
	q-2006.1-F	TATGTTTGGAGAGGGTGAATGG
	q-2006.1-R	CCCATCTGATCAAGCTTCTCTAC
	q-2013.1-F	TGAGGAGGGTGATTGCATTCCTCCT
	q-2013.1-R	GTGCAGAAAGGTCGAACAAAG
	q-3009.1-F	GCTCTTCTGGAAGGTGTCATAG
	q-3009.1-R	TCAGGAAAGGCTGCGAATAC
	q-3010.1-F	GCTCATCCTTACCTGTGACTAC
	q-3010.1-R	GACACTCCATTCACCTGACTAC
	q-11040.1-F	ACGAGAAGAGAACAAGGAAACA
	q-11040.1-F	CACCCAGACTCTTAGCGTATTT
Prokaryotic expression and protein purification	pETSUMO-Nb1L-F	ACAGATTGGTGGTAGACAAGCTATGGCCGGAGATTTTTGCCGC
	pETSUMO-Nb1L-R	CTTTGCGCCGAATAAATACCTATTATCTCTGACTTCTACGGTT

Application	Primer Name	Sequence (5'-3')
Prokaryotic expression and protein purification	pETSUMO-det-F	TGGACATGGAGGATAACG
	pETSUMO-det-R	CTAGCATAACCCCTTGGGGCCTCTAAACGGGTCTTGAGGGGTTTTTTG
	GST-Nb1L-F	GTTCTGTTCCAGGGGCCCCTGGGATCCATGGCCGGAGATTTTTGC
	GST-Nb1L-R	GAGTCGACCCGGGAATTCCGGGGATCCTTATCTCTGACTTCTACGGTTGGA
	GST-det-F	GGGCTGGCAAGCCACGTTTGGTG
	GST-det-R	CCGGGAGCTGCATGTGTCAGAGG
Mutant construction	pART27-Nb1L-F	CAAGGAATTCAGGCCTCCCATGGCCGGAGATTTTTGCCG
	pART27-Nb1L-R	CTCTAGATTAGGTACCCCCTTATCTCTGACTTCTACGGT
	pART27-detF	CACTCACGGCATGGA
	pART27-detR	CAAATATCATGCGATC
	det-PART1L-F	CACAGTCGATGAATCCAGGA
	det-PART1L-R	CCGAGAAAGTATCCATCATG
	det/cas9-F	GCAACGCTCTGTCATCGTTACAAT
	det/cas9-R	GCGATTAAGTTGGGTAACGCCAGGG
	56572_0@1(+)	CAGTGGTCTCATGCACTCCAAGTGATGGACTTAGTGTTTTAGAGC
	56572_0@1(-):	CAGTGGTCTCAAAACGCGGCCAAAATCTCCGGCCATG

Application	Primer Name	Sequence (5'-3')
RT-qPCR analysis of viral RNA accumulation in mutants	PVY-O-qpcr-1F	GGAGGAAGCAGCAAGAAAGA
	PVY-O-qpcr-1R	CCAGATGTACCAGCATTCACA
	PVY-NTN-qpcr-2F	CACAAGAGGAGAACACAGAGAG
	PVY-NTN-qpcr-2R	TCCGGAGAGACACTACATCA
m^6A-IP-qPCR	PVY-O-MeRIP-qPCR-1F	CGTGTGCTCAAGAACGGTAC
	PVY-O-MeRIP-qPCR-1R	CACAATACTAGGAGCGGCCA
	PVY-O-MeRIP-qPCR-2F	CGCATACACATTGCGTACAG
	PVY-O-MeRIP-qPCR-2R	GACTTCTGAATTGGCTCTGC
	PVY-O-MeRIP-qPCR-3F	CCTGAGAGAGAGCTCGAACT
	PVY-O-MeRIP-qPCR-3R	CTCTCAAGCCTCTCATGAGC
	PVY-O-MeRIP-qPCR-4F	GCCTTTGATGGATGCGTATG
	PVY-O-MeRIP-qPCR-4R	CGTGCACTTGCAGGTAGATA
	PVY-N-MeRIP-qPCR-1F	FCGTTTGCTTATTTCCAAGC
	PVY-N-MeRIP-qPCR-1R	GCATCCTTCGTCGATGAGAC
	PVY-N-MeRIP-qPCR-2F	GCTTGTTAAATAGAGAAGC
	PVY-N-MeRIP-qPCR-2R	GCCATGCACTTGCAGATAA
	PVY-N-MeRIP-qPCR-3F	GCAAAGAGAAGATACTTGC
	PVY-N-MeRIP-qPCR-3R	CCCAACCTCCATAAAACTT
	PVY-N-MeRIP-qPCR-4F	GCAAATTGACATCTCAAATAC
	PVY-N-MeRIP-qPCR-4R	CCATCATAACCCAAACTCC

Application	Primer Name	Sequence (5'-3')
Yeast two-hybrid (Y2H)	T7-det-F	ATACGACTCACTATAGGGCG
	3BD-det-R	CGTTTTAAAACCTAAGAGTC
	3AD-det-R	AGATGGTGCACGATGCACAG
	BK-1L-gene-F	CATGGAGGCCGAATTCCCGGGGATGGCCGGAGATTTTTGCCG
	BK-1L-gene-R	GCAGGTCGACGGATCCCCGGGAATTATCTCTGACTTCTACGGT
	AD-P1-gene-F:	GGCCAGTGAATTCCACCCGGGTATGGCAACTTACACATCAACAATC
	AD-P1-gene-R	CCCGTATCGATGCCCACCCGGGTGCTATTGAGTAACCTTGGAACGTGCAT
	AD-HC-Pro-gene-F:	GGCCAGTGAATTCCACCCGGGTATGGGGGTTATGGATTCAATGGTTCAG
	AD-HC-Pro-gene-R:	CCCGTATCGATGCCCACCCGGGTGCTATTGTCTCTCATTAATCCACAACCT
	AD-P3-gene-F:	GGCCAGTGAATTCCACCCGGGTATGTCCATAGCTATGATAGC
	AD-P3-gene-R:	CCCGTATCGATGCCCACCCGGGTGCTACTGGTGTCGCACATCATATT
	AD-PIPO-gene-F	GGCCAGTGAATTCCACCCGGGTGGAAAAAAATTATCTAAATC
	AD-PIPO-gene-R	CCCGTATCGATGCCCACCCGGGTGCTGGTGTCGCACATCATATTC
	AD-6K1-gene-F:	GGCCAGTGAATTCCACCCGGGTATGCGTTCCACACCAGGTGTTAAA
	AD-6K1-gene-R:	CCCGTATCGATGCCCACCCGGGTGCTACTGATGTCTAACTTCGTGGTC
	AD-CI-gene-F	GGCCAGTGAATTCCACCCGGGTATGTCCTTAGACGATGTGATCAAG

Application	Primer Name	Sequence (5'-3')
Yeast two-hybrid (Y2H)	AD-CI-gene-R:	CCCGTATCGATGCCCACCCGGGTGCTATTGGTGATGAACGAACTGC AAAG
	AD-6K2-gene-F	:GGCCAGTGAATTCCACCCGGGTATGGCTGCGACGTCACTTGCAAA G
	AD-6K2-gene-R:	CCCGTATCGATGCCCACCCGGGTGCTATTGGTGAGACACAGTCTCA ACTG
	AD-VPG-gene-F:	GGCCAGTGAATTCCACCCGGGTATGGGAAAAATAAATCCAAAAG
	AD-VPG-gene-R:	CCCGTATCGATGCCCACCCGGGTGCTATTCATGCTCCACTTCCTG
	AD-NIA-gene-F:	GGCCAGTGAATTCCACCCGGGTATGGCCAAATCACTCATGAGA
	AD-NIA-gene-R:	CCCGTATCGATGCCCACCCGGGTGCTATTGCTCTACAACAACATCA TG
	AD-NIB-gene-F	:GGCCAGTGAATTCCACCCGGGTATGGCTAAACATTCTGCGTGG
	AD-NIB-gene-R:	CCCGTATCGATGCCCACCCGGGTGCTATTGATGGTGCACTTCATAA GTATCG
	AD-CP-gene-F:	GGCCAGTGAATTCCACCCGGGTATGGGAAATGACACAATCGAT
	AD-CP-gene-R:	CCCGTATCGATGCCCACCCGGGTGCTACATGTTCTTGACTCCAAGT

Application	Primer Name	Sequence (5'-3')
Bimolecular Fluorescence Complementation (BiFC) and Subcellular Localization	pCV-cYFP-1L-gene-F	ACGCGTCGACATGGCCGGAGATTTTTGCCGCAGG
	pCV-cYFP-1L-gene-R	TCCCCCGGGTCTCTGACTTCTACGGTTGGAAGA
	pCV-nYFP-CI-gene-F	ACGCGTCGACATGTCCTTAGACGATGTGATCAAG
	pCV-nYFP-CI-gene-R	TCCCCCGGGTTGGTGATGAACGAACTGCAA
	pCV-nYFP-F	ACAACAGCCACAACGTCTAT
	pCV-cYFP-F	AACCACTACCTGAGCTACCAG
	pCV-YFP-R	AATCATCGCAAGACCGGC
	PCV-eGFP-det-F：	CCTTCGCAAGACCCTTCCTCT
	PCV-eGFP-det-3R	TTTACGTCGCCGTCCAGCTCG
	PCV-eGFP-1L-gene-F:	CTGCCCGGGGCCTGGGGTACCATGGCCGGAGATTTTTGCCG
	PCV-eGFP-1L-gene-3R	CATGTCGACGCACAGGGTACCTCTCTGACTTCTACGGTTGG
	PCV-eGFP-CI-gene-F:	CTGCCCGGGGCCTGGGGTACCATGTCCTTAGACGATGTGATCAAG
	PCV-eGFP-CI-gene-R	CATGTCGACGCACAGGGTACCTTGGTGATGAACGAACTGCAA
