# Supplementary figures and images for: ALKBH1L Is an m6A Demethylase and Mediates PVY Infection in Nicotiana benthamiana Through m6A Modification
Source: Plants (Basel). 2025 Dec 13;14(24):3796. doi: 10.3390/plants14243796 (PMC12736721; doi:10.3390/plants14243796)

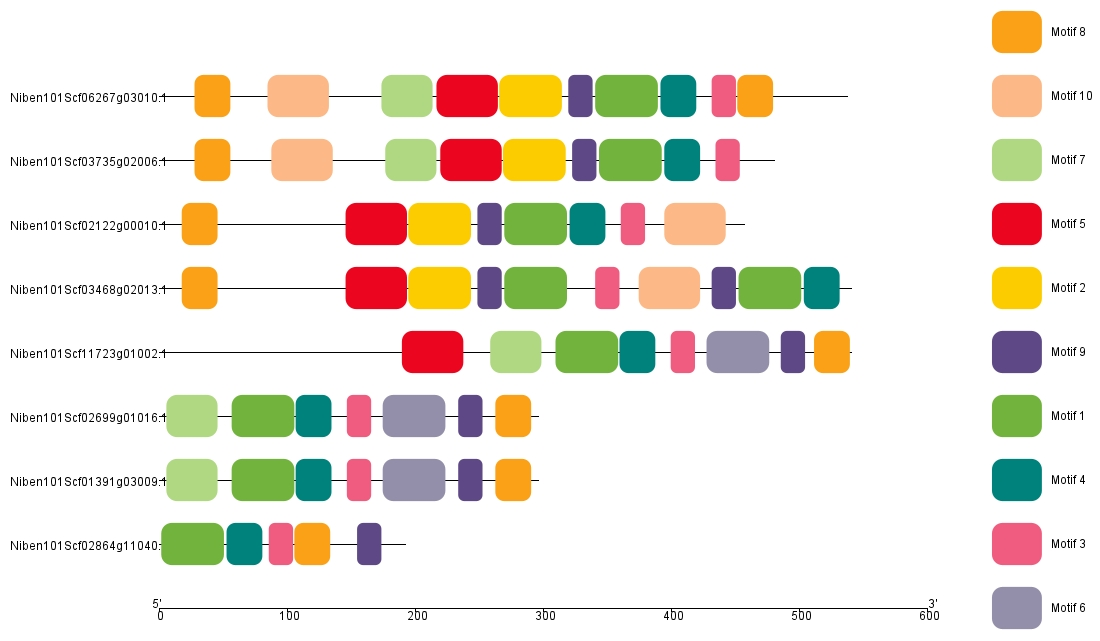

Supplement: Supplementary file 1 [file plants-14-03796-s001.zip › figure S1.tif]

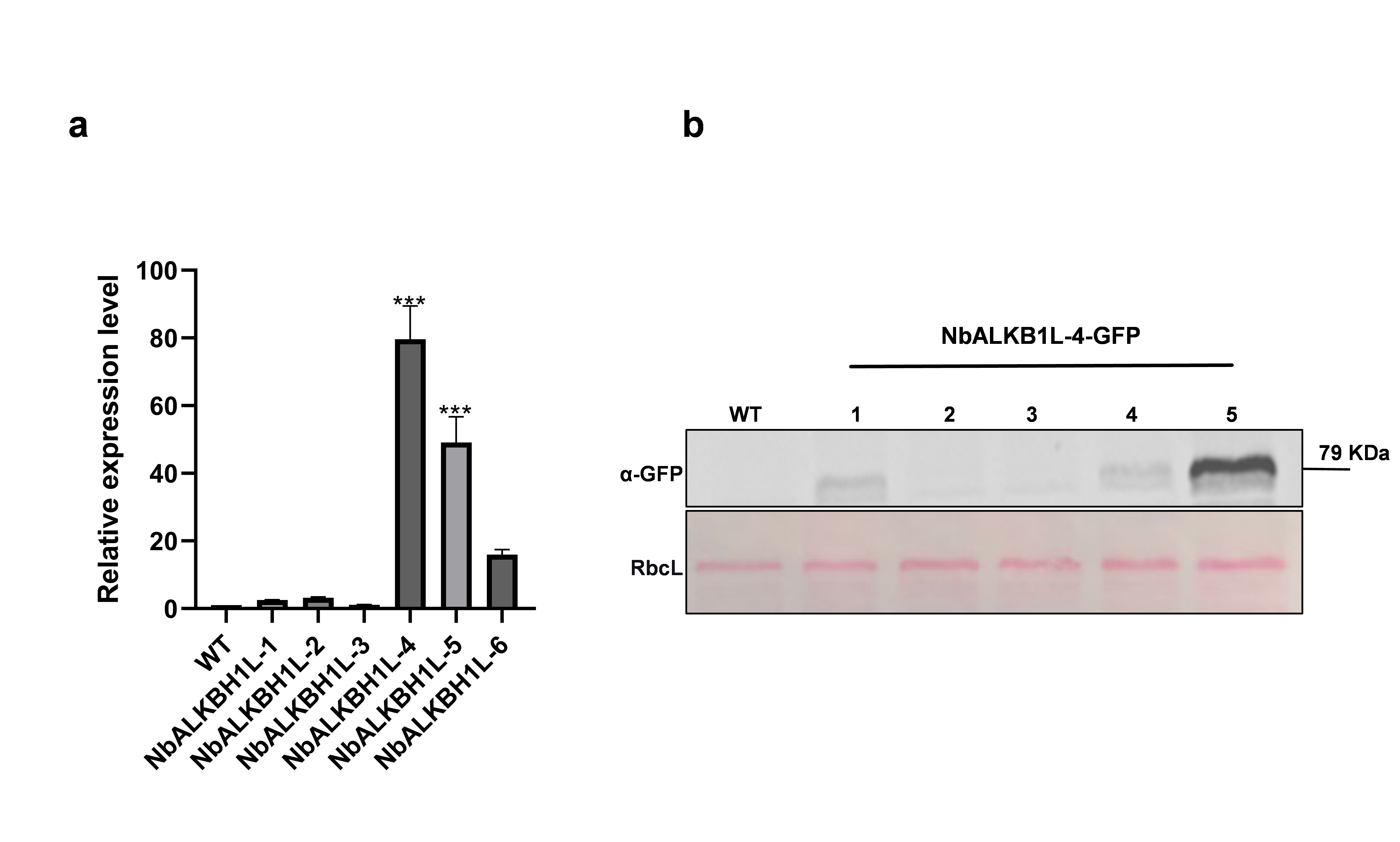

Supplement: Supplementary file 1 [file plants-14-03796-s001.zip › figure S2.tif]

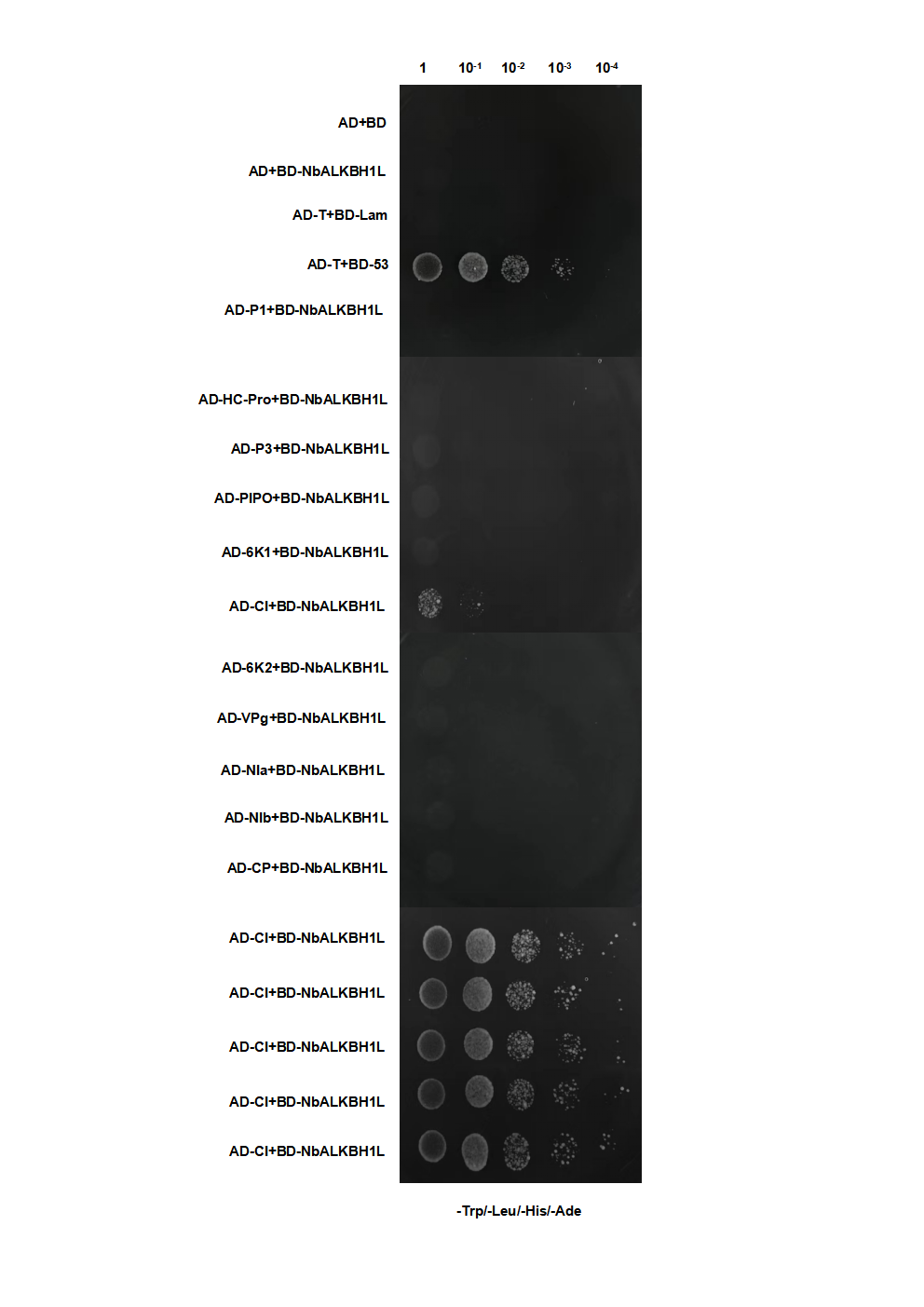

Supplement: Supplementary file 1 [file plants-14-03796-s001.zip › figure S3.tif]
